# A miRNA Host Response Signature Accurately Discriminates Acute Respiratory Infection Etiologies

**DOI:** 10.3389/fmicb.2018.02957

**Published:** 2018-12-11

**Authors:** Gregory D. Poore, Emily R. Ko, Ashlee Valente, Ricardo Henao, Kelsey Sumner, Christopher Hong, Thomas W. Burke, Marshall Nichols, Micah T. McClain, Erich S. Huang, Geoffrey S. Ginsburg, Christopher W. Woods, Ephraim L. Tsalik

**Affiliations:** ^1^Department of Biomedical Engineering, Duke University, Durham, NC, United States; ^2^Center for Applied Genomics and Precision Medicine, Duke University School of Medicine, Durham, NC, United States; ^3^Department of Hospital Medicine, Duke Regional Hospital, Durham, NC, United States; ^4^Division of Infectious Diseases, Duke University School of Medicine, Durham, NC, United States; ^5^Medicine Service, Durham VA Medical Center, Durham, NC, United States; ^6^Department of Biostatistics and Bioinformatics, Duke University School of Medicine, Durham, NC, United States; ^7^Duke Clinical and Translational Science Institute, Durham, NC, United States; ^8^Emergency Medicine Service, Durham VA Health Care System, Durham, NC, United States

**Keywords:** personalized medicine, micro RNA, transcriptome, respiratory tract infections, molecular diagnostics, host-pathogen interaction, bacterial infections, viral infections

## Abstract

**Background:** Acute respiratory infections (ARIs) are the leading indication for antibacterial prescriptions despite a viral etiology in the majority of cases. The lack of available diagnostics to discriminate viral and bacterial etiologies contributes to this discordance. Recent efforts have focused on the host response as a source for novel diagnostic targets although none have explored the ability of host-derived microRNAs (miRNA) to discriminate between these etiologies.

**Methods:** In this study, we compared host-derived miRNAs and mRNAs from human H3N2 influenza challenge subjects to those from patients with *Streptococcus pneumoniae* pneumonia. Sparse logistic regression models were used to generate miRNA signatures diagnostic of ARI etiologies. Generalized linear modeling of mRNAs to identify differentially expressed (DE) genes allowed analysis of potential miRNA:mRNA relationships. High likelihood miRNA:mRNA interactions were examined using binding target prediction and negative correlation to further explore potential changes in pathway regulation in response to infection.

**Results:** The resultant miRNA signatures were highly accurate in discriminating ARI etiologies. Mean accuracy was 100% [88.8–100; 95% Confidence Interval (CI)] in discriminating the healthy state from *S. pneumoniae* pneumonia and 91.3% (72.0–98.9; 95% CI) in discriminating *S. pneumoniae* pneumonia from influenza infection. Subsequent differential mRNA gene expression analysis revealed alterations in regulatory networks consistent with known biology including immune cell activation and host response to viral infection. Negative correlation network analysis of miRNA:mRNA interactions revealed connections to pathways with known immunobiology such as interferon regulation and MAP kinase signaling.

**Conclusion:** We have developed novel human host-response miRNA signatures for bacterial and viral ARI etiologies. miRNA host response signatures reveal accurate discrimination between *S. pneumoniae* pneumonia and influenza etiologies for ARI and integrated analyses of the host-pathogen interface are consistent with expected biology. These results highlight the differential miRNA host response to bacterial and viral etiologies of ARI, offering new opportunities to distinguish these entities.

## Background

The rapid rise of antibiotic-resistant infections in the last two decades has triggered alarm within global healthcare systems (Spellberg et al., 2008). In December 2014, one report estimated nearly 10 million additional annual deaths due to antimicrobial resistance by 2050, costing the global economy up to $100 trillion in lost output (O’Neill, 2016). ARIs are a major contributor to this problem in the U.S., accounting for roughly 40% of all antibiotics prescribed in the adult outpatient setting (Shapiro et al., 2014), approximately half of which are deemed unnecessary because viral etiologies cause the majority of these infections (Fleming-Dutra et al., 2016). Clinical providers struggle with assessing the probability of a bacterial infection when symptoms between bacterial and viral infections are very similar. Providing clinicians with diagnostic tools to quickly and reliably distinguish viral from bacterial infections in ARI could significantly reduce antibiotic prescriptions.

Host-based diagnostics offers a mechanism to fill this void. For example, the peptide biomarker procalcitonin has successfully reduced antibiotic prescriptions for ARI based on its preferential rise in bacterial infection (Schuetz et al., 2012). However, there are limitations to this biomarker including false positives in non-infectious conditions as well as false negatives early in disease and patients with atypical bacterial infections (Berg and Lindhardt, 2012; Giannakopoulos et al., 2017; Self et al., 2017). Moreover, nearly a quarter of patients with typical bacterial infection had a low procalcitonin value that would have inappropriately restricted antibacterial use (Self et al., 2017). As a result, there remains a need to improve the differentiation of viral and bacterial infection, particularly in the context of ARI. Numerous host transcriptional signatures have demonstrated a remarkable ability to make this distinction with results that are significantly better than procalcitonin (Suarez et al., 2015; Tsalik et al., 2016). Use of host response biomarkers that incorporate a group of expressed gene transcripts to classify bacterial from viral infections holds tremendous potential to bridge this gap. However, many transcripts are needed to classify the etiology of ARI particularly when non-infectious etiologies, a necessary control group, are considered. Given the technical challenges associated with rapid, quantitative or semi-quantitative multi-plexing of mRNA concentrations, we considered alternative host response targets. Furthermore, better discrimination may ultimately come from combining different types of host response measurements (e.g., microRNA, mRNA, proteomics, metabolomics, etc.) using a multimodal approach.

MicroRNAs (miRNAs) are attractive targets for a multimodal approach because they are highly conserved and poised at the top of cellular regulatory networks. miRNAs are a family of short, non-coding RNAs (usually 19-25 nt) that regulate cellular gene expression via degradation or translational repression of their targeted mRNA transcripts (Bartel, 2009). Furthermore, miRNAs have structural properties that are desirable for diagnostic quantification: resistance to boiling; freeze-thaw; presence in serum and tissues; and slow rates of decay (Gilad et al., 2008; Mitchell et al., 2008). Circulating human miRNAs demonstrate differential expression in response to infection (Correia et al., 2017). However, the study of miRNAs is relatively new compared to that of mRNA analysis (Bartel, 2009; Correia et al., 2017) and translating this to human disease is still in its infancy. Within infectious disease, human samples have been employed in the study of *Mycobacterium tuberculosis* (Fu et al., 2011; Qi et al., 2012; Abd-El-Fattah et al., 2013; Zhang et al., 2013; Zhang H. et al., 2014), sepsis (Wang et al., 2010, 2013; Roderburg et al., 2013; Benz et al., 2015), hepatitis viruses (Zhang et al., 2010, 2012; Xu et al., 2011; Arataki et al., 2013; Zhang X. et al., 2014), influenza (Song et al., 2013; Tambyah et al., 2013), and dengue (Ouyang et al., 2016; Tambyah et al., 2016), but often times yield inconsistencies that may be related to differences in sample type, sample collection, data collection, and analysis. Moreover, published studies have focused on the miRNA response to one particular infectious state, but few compare disease states to each other. Thus, inconsistent data among studies and the lack of clinically useful comparison groups have made development of miRNA biomarkers for infectious disease challenging.

This study explores the miRNA response to both viral and bacterial etiologies of ARI compared to the healthy state, but it also defines the differential response between the two types of infection. Specifically, we compared miRNA and mRNA expression in whole blood of adults with community onset *Streptococcus pneumoniae* pneumonia (Glickman et al., 2010; Tsalik et al., 2010) to those from an experimental influenza challenge study representing healthy (pre-inoculation) and H3N2 influenza infected patients (post-inoculation) (Liu et al., 2016; McClain et al., 2016). Using these data, we generated diagnostic miRNA signatures to accurately discriminate *S. pneumoniae* bacterial infection, influenza H3N2 viral infection, and the healthy state using penalized logistic regression models. DE mRNAs from the same subjects were employed to computationally predict miRNA:mRNA interactions using negative correlation analysis. The integration of these two data types informed the regulatory networks at the host–pathogen interface and provided biological plausibility that the identified miRNAs are indeed important mediators of the host response to viral and bacterial infection.

## Materials and Methods

### Study Design

Studies were approved by relevant Institutional Review Boards and in accordance with the Declaration of Helsinki. All subjects or their legally authorized representatives provided written informed consent.

H3N2 Influenza Human Challenge Cohort: Healthy adults were recruited for experimental challenge with influenza A/Wisconsin/67/2005 (H3N2) (DEE5 study, *n* = 21) (Liu et al., 2016; McClain et al., 2016). Subjects at baseline, prior to influenza challenge, served as the healthy controls. Following exposure, thirteen subjects developed symptomatic influenza infections while eight remained asymptomatic. For symptomatic patients (13), the time of maximal symptoms averaged 67 h following exposure (range, 50–114 h) and the PAXgene Blood RNA (PreAnalytix; Franklin Lakes, NJ, United States) tube nearest to this time was selected for analysis. Detailed methods of the inoculation, viral titers employed, and subsequent processing of the RNA samples have been described (Zaas et al., 2009, 2013; Liu et al., 2016; McClain et al., 2016).

Bacterial *Streptococcus pneumoniae* Cohort: For this pilot project, ten adult subjects were selected from the larger Community Acquired Pneumonia and Sepsis Outcome Diagnostic (CAPSOD) study, which focused on patients with community-onset, suspected sepsis (ClinicalTrials.gov NCT00258869) (Glickman et al., 2010; Tsalik et al., 2010). Subjects presented to the ED at one of three participating hospitals (Duke Hospital, UNC-Chapel Hill, and Henry Ford Hospital) where they were enrolled and samples collected. Cases were defined as a clinical syndrome consistent with pneumonia along with the identification of *S. pneumoniae* as the etiologic agent by culture (respiratory or blood sample) or with urinary antigen testing. This particular subset of CAPSOD was chosen because they were the only cases with *S. pneumoniae* pneumonia with a remaining PAXgene Blood RNA tube available for analysis.

### Procalcitonin Measurements

Procalcitonin (PCT) was measured for the nine subjects in the *S. pneumoniae* cohort who had an available sample using the B⋅R⋅A⋅H⋅M⋅S PCT sensitive KRYPTOR assay (Thermo Fischer Scientific).

### Generation and Normalization of Transcriptomic Data

Total RNA was extracted from whole blood using Qiagen’s PAXgene Blood mRNA Kit or miRNA Kit (Hilden, Germany) according to manufacturer specifications. Total RNA for mRNA and miRNA were extracted separately from two different PAXgene tubes, one extraction designated for either miRNA or mRNA analysis. Samples were screened for RNA quantity and quality using a NanoDrop Spectrophotometer (Thermo Scientific; Waltham, MA, United States) and Agilent 2100 Bioanalyzer (Santa Clara, CA, United States).

The transcriptional response was investigated using Affymetrix Arrays of mRNA. Hybridization and data generation for Affymetrix U133 Plus 2.0 Arrays were performed in accordance with the manufacturer’s recommended protocol.

Analysis of the miRNA response by RNA sequencing at the Duke University Sequencing and Genomic Technologies Shared Resource was accomplished using Illumina’s TruSeq Small RNA sample preparation kit (Illumina; San Diego, CA, United States). Read depth was approximately 15 M reads per sample (50 bp single-end read). The resultant data were filtered through quality control measures (FastQC) (Andrews, 2010), had adapter-ends and poor quality bases trimmed (Trimmomatic) (Bolger et al., 2014); and then evaluated using the miRDeep2 algorithm (Friedlander et al., 2012) for read collapsing, mapping, and quantification using default parameters. miRDeep2 generated a number of potential novel miRNA transcripts that were scored as part of the standard processing pipeline. However, to ensure our analysis was only performed using miRNAs with confirmed biological activity, these potential novel miRNA transcripts were excluded from signature development. All miRNA data were generated in a single RNA sequencing batch. Discrete counts were normalized using a supervised, regularized, log transformation (Love et al., 2014).

The corresponding mRNA Affymetrix microarray data was generated as part of two prior independent studies (Woods et al., 2013; Tsalik et al., 2016). Those two independent mRNA microarray batches were completely confounded by phenotype such that all subjects from the influenza challenge study had mRNA expression data generated in one batch and all subjects with community onset *S. pneumoniae* pneumonia were generated in another microarray batch. As a result of confounding by batch, this mRNA microarray data could not be used for modeling and signature development. However, we correlated the changes in mRNA transcription that occurred in response to changes in miRNA expression. To do this, we normalized for these two batches. The second batch including community onset pneumonia also included subjects with community onset influenza. Although these were not the same subjects in the influenza challenge, we assumed they would have similar gene expression changes and therefore used them as a normalizing control using the robust multiarray average (RMA) method (Bolstad et al., 2003; Irizarry et al., 2003). Affymetrix probes were then filtered using the genefilter R package to identify Affymetrix probes associated with an Entrez ID and target gene. After the data had been normalized among batches and Affymetrix probes had been filtered, batch correction was completed by employing the SNM method (Mecham et al., 2010), ComBat (Johnson et al., 2007), and fRMA (McCall et al., 2010). These methods of batch correction were compared using PVCA (Bushel, 2013). SNM demonstrated the best performance at removing batch variance and was used for subsequent analysis.

### Multivariable Statistical Analysis

Hypothesis testing was performed separately for miRNA and mRNA data. For miRNA data, the empiric variance of the data informed dispersion estimates and size factors, which were subsequently used to fit negative binomial generalized linear models following standard DESeq2 protocol (Love et al., 2014). Wald parametric testing was then utilized to infer the true population of DE miRNAs. Multiple testing correction was performed with the Benjamini–Hochberg method (Benjamini and Hochberg, 1995) and a FDR was calculated using the stringent requirement that a transcript be present in all subjects. Unlike the prior comparisons, the viral vs. healthy comparison used the same individuals before and after infection and was analyzed as a paired dataset, requiring that the transcript be present in three-quarters of subjects.

For mRNA data, fold changes and standard errors were estimated by fitting a generalized linear model to each Affymetrix probe. Empirical Bayesian statistics were then used to smooth the standard errors of the estimated log-fold changes [*limma* modeling (Smyth, 2005)]. Like the miRNA analysis, multiple testing correction was performed at a FDR of less than or equal to 1% (*Q* ≤ 0.01). Significantly overrepresented pathways were determined using gene ontologies in the DAVID (Huang et al., 2007).

### miRNA Host–Response Signature Development

Penalized, sparse logistic regression was performed on miRNA data to determine diagnostic signatures for bacterial infection, viral infection, and healthy, similar to prior work focusing on mRNA (Tsalik et al., 2016). Normalized expression values of the miRNA signatures were also compared between infectious etiologies using Welch Two-Sample *t*-tests with a significance level of *p* ≤ 0.01. Least absolute shrinkage and selection operator (LASSO or L1) regularization was applied to find the smallest signature while maintaining diagnostic accuracy (Friedman et al., 2010). To estimate optimal model parameters and compute classification performance, nested LOOCV was implemented. Nested LOOCV was performed to optimize the sparsity-inducing parameter of the logistic regression model and improve classification performance. ROC curves were then generated with the ROCR package in R (Sing et al., 2005). The optimal probability threshold was then determined by minimizing the Euclidean distance from the upper left point (0,1) to the ROC curve and used for subsequent confusion matrix generation. Sensitivity, specificity, and overall classification accuracy were computed to assess classification success.

### Generating miRNA:mRNA Interaction Networks

miRNAs that discriminated between relevant clinical groups in the models were used for integrated analysis with DE mRNA. These two groups of miRNAs and mRNAs were considered to be biologically linked if they met two criteria. First, suspected miRNA binding sites in the mRNA of interest must be found within the top 20% of computationally predicted scores in at least two of the following eight major public databases [TargetScan (Lewis et al., 2005; Agarwal et al., 2015), PITA (Kertesz et al., 2007), PicTar (Krek et al., 2005), miRDB (Wong and Wang, 2015; Wang, 2016), miRanda (Enright et al., 2003; John et al., 2004; Betel et al., 2008, 2010), MicroCosm (formerly miRBase) (Griffiths-Jones, 2006; Griffiths-Jones et al., 2006, 2008; Kozomara and Griffiths-Jones, 2011; Kozomara and Griffiths-Jones, 2014), ElMMo(Hausser et al., 2009), DIANA-microT-CDS (Kiriakidou et al., 2004; Maragkakis et al., 2009)]. Since it is known that miRNA binding leads to a down regulation of genes via targeted destruction or translational repression, the second criteria was that the miRNA:mRNA pair must be significantly, negatively correlated (H_A_: correlation value is less than 0, *Q* ≤ 0.01). Pearson correlation coefficients were calculated using the miRComb package in R (Vila-Casadesus et al., 2016). Significantly correlated, predicted miRNA:mRNA pairs were then used for network visualization in Cytoscape (Shannon et al., 2003).

## Results

### Cohort Design and Clinical Characteristics

**FIGURE 1:**
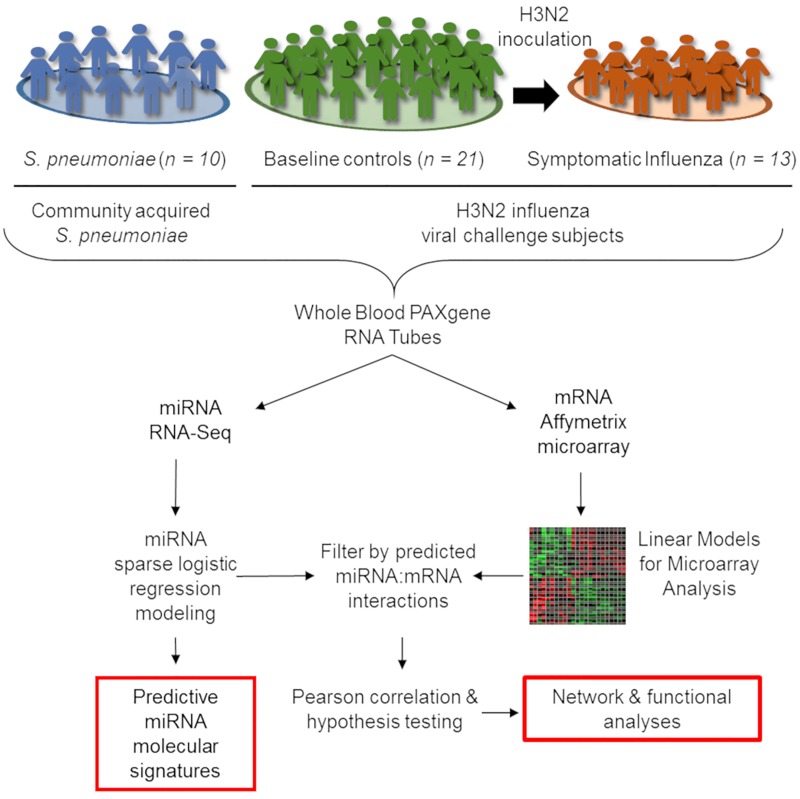
Outline of integrative transcriptomic analysis to create and internally validate miRNA signatures. Forty four whole-blood RNA samples were included in this analysis, comprising 21 healthy controls, 13 symptomatic influenza subjects, and 10 subjects with community-onset *S. pneumoniae* pneumonia. Transcriptional expression measured using miRNA RNA-Seq and mRNA Affymetrix microarrays were utilized for subsequent analysis. After data normalization, LASSO-penalized sparse logistic regression generated miRNA signatures while linear models for microarray analysis (*limma)* identified differentially expressed mRNAs. Interaction between signature miRNA and differentially expressed mRNAs in each comparison were explored by first identifying a potential interaction in publicly available databases and then using negative Pearson correlation to identify miRNA:mRNA pairs with correlation of expression consistent with known biology.

We report a retrospective, pilot analysis to explore the ability of circulating miRNAs from whole blood to differentiate ARI due to bacterial and viral pathogens. Ten subjects with community acquired pneumococcal pneumonia (Glickman et al., 2010; Tsalik et al., 2010) were compared to 21 healthy controls and 13 symptomatic H3N2 influenza cases (Liu et al., 2016; McClain et al., 2016). Given the small size of this pilot study, groups were intentionally selected to be homogenous for a single pathogen representative of ARIs to allow the best opportunity to detect a statistically significant signal between the bacterial, viral, and healthy groups. Figure 1 depicts cohort selection and experimental design.

Due to differences in study design between CAPSOD and the influenza challenge, subject demographics differed between the clinical groups. Influenza challenge study subjects were younger, disproportionately caucasion, and had similar numbers of male and female subjects. The CAPSOD study population was older, had a larger proportion of African Americans, and had more females than males (Table 1).

**Table 1:** Summary of patient and clinical characteristics.

	miRNA signature generation		
Characteristics	Influenza A (H3N2) Challenge Study		CAPSOD Study
	Healthy baseline	Symptomatic influenza	Bacterial infection
Number of samples	21	13	10
Age (mean (95% confidence), range)	26 (2.81), 20-44	27.62 (4.20), 20-44	50.9 (10.91), 19-76
**Sex (number of subjects)**			
Male	12	6	3
Female	9	7	7
**Race (number of subjects)**			
American Indian/Alaska Native	0	0	0
Asian	2	1	0
Black/African American	1	1	7
White	17	10	3
Other	1	1	0
Unknown	0	0	0


### miRNA Host–Response Signature Accurately Discriminates ARI Etiologies

**FIGURE 2:**
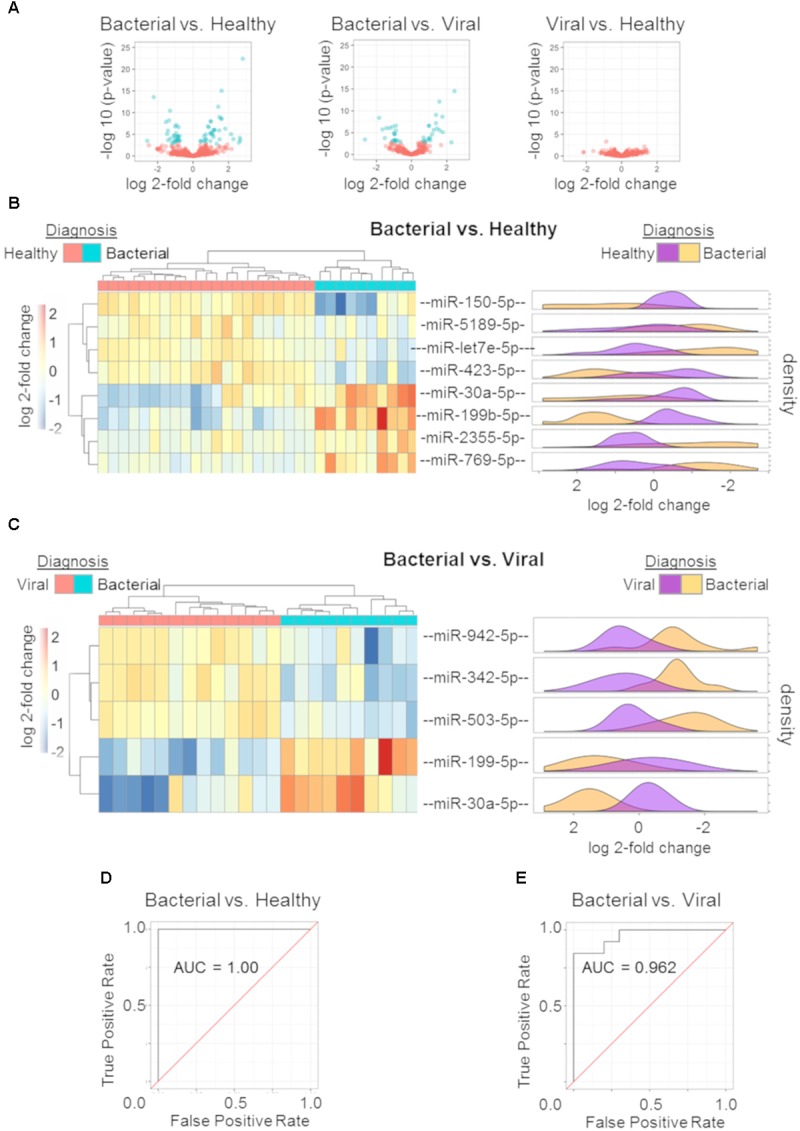
miRNA signature and performance estimates using cross validation. Volcano plots of the three comparisons using miRNA expression data: bacterial vs. healthy (left); bacterial vs. viral (middle); viral vs. healthy (right). All markers in blue have a FDR of ≤ 1% (Q ≤ 0.01) **(A)**. Regularized logistic regression modeling was performed for each comparison yielding discriminating miRNA signatures for bacterial vs. healthy and bacterial vs. viral. Hierarchical clustering with *post hoc* labeling was performed and signature miRNAs are represented for bacterial vs. healthy **(B)** and bacterial vs. viral **(C)** comparisons. Normalized expression values after regularized log transformation are shown as marginal density plots (in order) on the right **(B,C)**. ROC curve of bacterial vs. healthy signature **(D)** and the bacterial vs. viral signature **(E)** using a LOOCV protocol reveal an AUC of 1.00 and 0.962 respectively.

miRNA profiles were generated for subjects with *S. pneumoniae* pneumonia, influenza, and healthy subjects. Wald parametric testing identified a number of DE miRNAs (Figure 2A): *S. pneumoniae* infection (bacterial) vs. healthy (67 DE miRNAs) (Supplementary Table S1) and *S. pneumoniae* infection (bacterial) vs. H3N2 influenza (viral) infection (40 DE miRNAs) (Supplementary Table S2) with a stringent FDR of less than or equal to 1%. We found considerable overlap of miRNAs between these two comparisons (Supplementary Tables S1, S2). MiRNA-150-p and miRNA-96-5p revealed a Q-value of 11.7% for the influenza (viral) vs. healthy comparison, but did not meet the strict cutoff of FDR less than or equal to 1%.

Diagnostic miRNA signatures for bacterial vs. healthy and bacterial vs. viral were generated using L1 regularized logistic regression. The viral vs. healthy comparison did not demonstrate the ability to discriminate influenza infection from healthy (data not shown). Eight miRNAs contributed to the bacterial vs. healthy model. Four were upregulated (hsa-miR-30a-5p, hsa-miR-199b-5p, hsa-miR-2355-5p, and hsa-miR-769-5p) and four were downregulated (hsa-miR-150-5p, hsa-miR-5189-5p, hsa-let-7e-5p, and hsa-miR-423-5p) in bacterial infection compared to the healthy state. As few as five miRNAs could discriminate between bacterial and viral infection. Two were upregulated (hsa-miR-199b-5p and hsa-miR-30a-5p) and three were downregulated (hsa-miR-942-5p, hsa-miR-342-5p, and hsa-miR-503-5p) in *S. pneumoniae* infection compared to influenza infection. Hsa-miR-199b-5p and hsa-miR-30a-5p were common to the two models, both of which were upregulated in bacterial infection.

We sought to validate these signatures in other datasets. However, there was no publically available dataset including miRNA expression from both bacterial and viral infection. A validation dataset should have both groups in order to allow model training and to avoid confounding by batch. Instead, we used hierarchical clustering with complete linkage to assess leave-one-out cross validation (LOOCV) performance. In this unsupervised method, the algorithm does not know each subject’s outcome *a priori* and only separates them based on their Euclidean distances. The hierarchical clustering revealed excellent discrimination between *S. pneumoniae* infection vs. healthy (Figure 2B) as well as *S. pneumoniae* infection vs. influenza infection (Figure 2C). Normalized count distributions for each miRNA in the models was plotted as a function of clinical phenotype (right side of Figures 2B,C). All *p*-values for individual miRNAs in the signatures were below the significance threshold of 0.01 (Student *t*-test on Bacterial vs. Healthy signature ranges from *p* = 2.69e-07 to *p* = 0.0062 per miRNA; Bacterial vs. Viral signature ranges from *p* = 7.54e-07 to *p* = 0.0012 per miRNA). ROC curves based on nested LOOCV are shown (Figures 2D,E, respectively) along with their corresponding AUCs. The bacterial vs. healthy classifier yielded an AUC of 1.00 while the bacterial vs. viral classifier yielded an AUC of 0.962. These data reveal good performance of the miRNA expression model to discriminate bacterial from healthy and viral subjects for these pathogens, consistent with known differences in the host response to these different pathogen classes.

The ability to accurately classify bacterial and viral infections was assessed by determining overall accuracy, sensitivity, and specificity using LOOCV. Confusion matrices and their associated performance statistics are shown in Table 2. The bacterial vs. healthy model correctly classified samples with 100% accuracy (Acc > NIR, *p*-value = 5.709e-06). The bacterial vs. viral model had good performance as well, but misclassified two viral samples as bacterial, giving this model 91.3% overall accuracy (Acc > NIR, *p*-value = 3.367e-04). The models were highly sensitive (100% in both comparisons) and very specific (100% for bacterial vs. healthy and 84.6% for bacterial vs. viral) for this cohort. In order to determine if the signatures and their performance were robust to different statistical methods, we generated new models using Elastic Net. The signatures obtained using Elastic Net contained the same miRNAs found in the LASSO model and their classification performance was the same suggesting that the analysis method was not a significant contributor to the model’s content or performance.

**Table 2:** Confusion matrices and associated performance estimates of miRNA signatures.

		Bacterial vs. Healthy Signature				Bacterial vs. Viral Signature	
		Actual				Actual	
		Bacterial	Healthy			Bacterial	Viral
**Predicted**	Bacterial	21	0	**Predicted**	Bacterial	10	2
	Healthy	0	10		Viral	0	11
**Statistics**	ROC optimal threshold	0.648		**Statistics**		0.770	
	Accuracy (95% CI)	1.0000 (0.8878, 1.0000)				0.9130 (0.7196, 0.9893)	
	*P*-value (Acc > NIR)	5.709e-06				3.367e-04	
	Kappa	1.0000				0.8271	
	Sensitivity	1.0000				1.0000	
	Specificity	1.0000				0.8462	


We compared the miRNA classifier to the biomarker procalcitonin in our cohort. Procalcitonin has been shown to be elevated in bacterial infection (accepted clinical cut-off > 0.25 μg/liter) and low in viral or non-infection (accepted clinical cut-off ≤ 0.25 μg/liter) (Meisner, 2014). Since this study utilized retrospective samples, serum or plasma were available in nine of the ten bacterial subjects but were unavailable for healthy or viral subjects. Analysis of procalcitonin levels in available samples revealed only 66.7% (6 of 9) of subjects with *S. pneumoniae* infection had procalcitonin levels above > 0.25 μg/liter (Supplementary Table S3). Thus, sensitivity to detect bacterial infection in this small cohort is only 66.7% for procalcitonin compared to 100% for both host-response models.

We also determined whether there was an impact of gender on model performance. Stratifying data normalization by gender revealed no differences in predicted class probabilities based on paired Wilcoxon signed rank test between the bacterial vs. healthy (W statistic = 216; *p* = 0.5422) and bacterial vs. viral models (W statistic = 144; *p* = 0.8697). Due to the small cohort we were unable to evaluate the effects of age and race. However, prior meta-analysis of disease specific mRNA signatures, including infectious disease signatures, did not show significant differences due to age, sex, or race (Wang et al., 2016). Our evaluation of changes in the model due to sex and prior research indicate that these results are related to the host response to a pathogen rather than differences in demographics.

### Differential Expression and Functional Analysis of mRNAs in the ARI Host–Response

**FIGURE 3:**
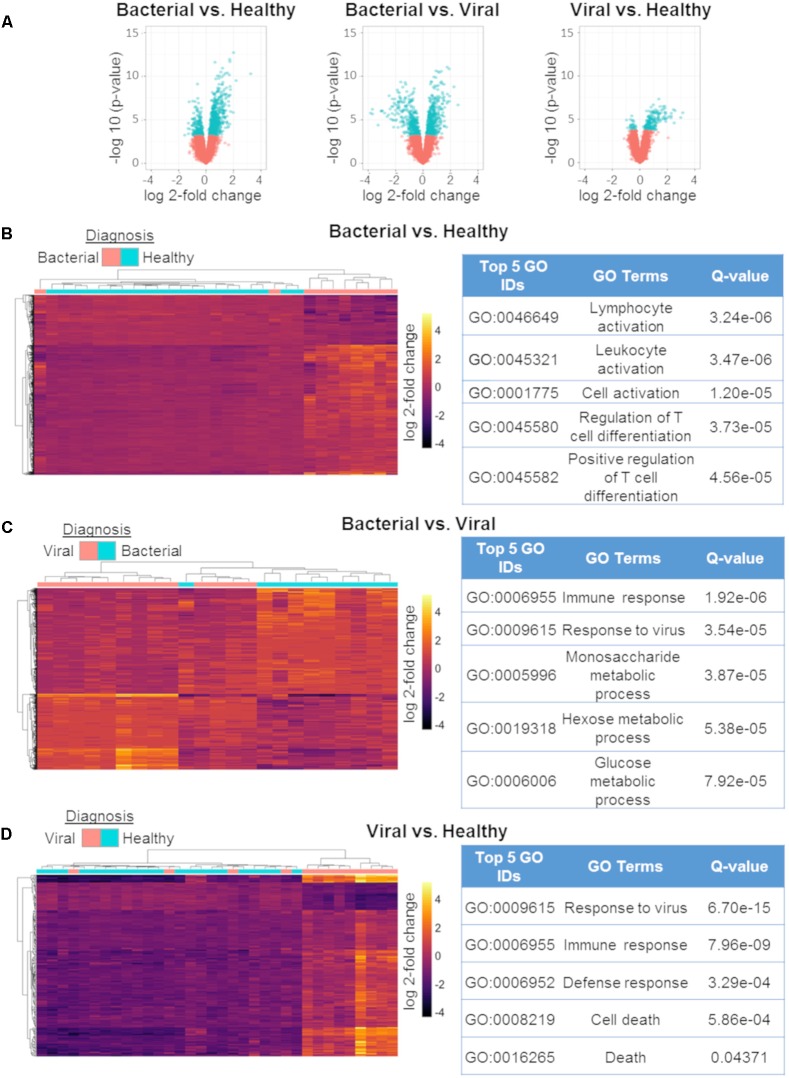
mRNA differential expression analysis using generalized linear models. Volcano plots of the three comparisons using mRNA expression data:bacterial vs. healthy (left);bacterial vs. viral (middle); viral vs. healthy (right). All markers in blue have a FDR of ≤ 1% (*Q*-value ≤ 0.01) **(A)**. Differentially expressed mRNAs between bacterial vs. healthy **(B)**, bacterial vs. viral **(C)**, and viral vs. healthy **(D)** and their associated top five GO results. All heat maps represent hierarchical clustering using only mRNAs found to have *Q* ≤ 0.01 with labeling of the phenotype performed *post hoc* [*n* = 802 for **(A)**, *n* = 786 for **(C)**, *n* = 201].

To explore the biological significance of the discovered miRNAs, we used matched mRNA expression data from the same subjects and timepoints. Generalized linear modeling with empirical Bayesian statistics to smooth the standard errors of log-fold changes (Smyth, 2005) was used to identify DE mRNAs between the three phenotypes. *S. pneumoniae* infection vs. influenza infection had 802 DE transcripts, *S. pneumoniae* infection vs. healthy had 786 DE transcripts, and influenza infection vs. healthy had 201 DE transcripts (Q ≤ 0.01) (Figure 3A). DE genes between the three comparisons were used to cluster samples, followed by *post hoc* labeling to demonstrate their utility in discriminating between etiologies of ARI (Figures 3B–D). Using these DE genes, functional analyses were then performed using gene ontologies in DAVID (Huang et al., 2007). The GO pathways that were enriched in these comparisons agreed with known biology and were highly significant (Figures 3B–D), such as Lymphocyte Activation, Immune Response, and Response to Virus. We compared the DE mRNAs for the viral vs. healthy comparison to previously published gene expression signatures, which revealed matches for 60 of the 201 DE transcripts (Zaas et al., 2009, 2013; Woods et al., 2013; McClain et al., 2016; Tsalik et al., 2016). Thus, the mRNA responses to bacterial or viral infection observed here were consistent with the expected host response to these infections.

### miRNA:mRNA Network Analysis Reveals Biologically Relevant Pathways

Having identified discriminating miRNAs and mRNAs, we examined miRNA expression in conjunction with patient-matched mRNA data and formed an integrative transcriptomic framework. Analysis focused on miRNAs in the signatures and DE mRNAs within each comparison (bacterial vs. viral, bacterial vs. healthy). We included miRNA-150-p (*p*-value = 0.020 by *t*-test) from the viral vs. healthy comparison because there is a known relationship to immune function.

**FIGURE 4:**
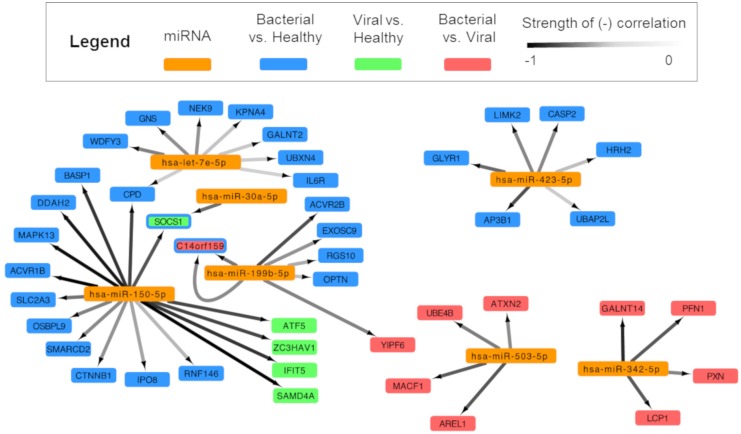
Integrated analysis of signature miRNAs and their mRNA interactions. miRNA and mRNA data for each subject were analyzed and were paired based on (1) a high probability of predicted miRNA:mRNA and (2) negatively correlated expression. Cytoscape was used to show an integrated transcriptomic framework. Although the viral vs. healthy miRNA signature had no statistically significant differentially expressed miRNAs, one approached that threshold and was included here (hsa-miR-150-5p). Arrows are drawn between significantly, negatively correlated miRNA:mRNA pairs where the darkness of the line denotes the strength of the correlation (range: –0.85, –0.5185). Genes represented are predicted to be regulated by miRNAs in the bacterial vs. healthy (blue), viral vs. healthy (green), and bacterial vs. viral (red) signatures. The SOCS1 gene is dually colored to represent the finding that miRNA-150 is a predicted regulator during viral infection (green) and miRNA-30a during bacterial infection (blue). miRNA-199b is overexpressed during bacterial infection and is associated with suppression of the gene C14orf159 in both the bacterial vs. healthy (blue) and bacterial vs. viral (red) comparisons; thus, this gene also is represented by two colors.

In order to build a miRNA:mRNA interaction network, we first identified potential miRNA:mRNA complementary binding pairs and then utilized inverse correlation analysis to identify pairs where the miRNA levels negatively correlated with the associated mRNA, commensurate with known biology. This is an approach successfully utilized by others (Gennarino et al., 2009; Lionetti et al., 2009). Pairs meeting the threshold of significance (*Q* ≤ 0.01, *n* = 46) were incorporated into network analysis (Figure 4).

After applying statistical filters, six miRNAs were paired with 43 mRNA targets. Hsa-miR-150-5p was linked to the greatest number of mRNA targets (sixteen), including SOCS1, SAMD4A, and IFIT5, which were part of previously published gene expression signatures for viral infection (Woods et al., 2013; Zaas et al., 2013; McClain et al., 2016). Three miRNAs from the *S. pneumoniae* infection vs. healthy comparison associated with mRNAs (hsa-let-7e-5p with eight mRNA targets; hsa-miR-423-5p with six mRNA targets, and hsa-miR-30a-5p with 1 mRNA target). Two miRNAs paired exclusively with mRNAs that were DE in the *S. pneumoniae* vs. influenza infection comparison (hsa-miR-503-5p and hsa-miR-342-5p with four mRNA targets each). To our knowledge, this forms the first integrative miRNA-mRNA transcriptomic model for the human host response to ARI. Not only are these results consistent with known miRNA biology, but they also reveal possible targets and regulatory pathways for miRNAs in the context of bacterial and viral infection diagnosis.

## Discussion

### Summary

We report a pilot study that identified miRNA host responses to bacterial and viral respiratory infections in subjects with pneumococcal pneumonia and H3N2 influenza infection. This revealed that distinct changes in miRNA expression occurred in response to these infections, discriminating subjects with *S. pneumoniae* infection from healthy subjects or those with influenza infection. We validated these results statistically by using leave-one-out cross validation and biologically by identifying miRNA:mRNA networks that recapitulated relevant pathways. These results demonstrate the value of diagnostic miRNA signatures to discriminate bacterial and viral etiologies of ARI, paving the way for prospective validation in larger cohorts.

### Host–Response miRNA Signatures as Biomarkers for ARI

Acute respiratory infections present an important challenge in the battle against antibiotic resistance. The goal of this work was to determine if the host miRNA response would offer similar classification accuracy as previously identified host mRNA signatures (Suarez et al., 2015; Tsalik et al., 2016). One feature of miRNAs that make them attractive biomarkers is their role as upstream regulators and their ability to orchestrate cellular pathways (Podshivalova and Salomon, 2013; Huang et al., 2015; Robertson and Ghazal, 2016). Thus, a single miRNA may be sufficient to detect changes in many important cellular signaling pathways, limiting the number of probes needed for a host response transcriptional biomarker. For example, viral infection induces many interferon pathway genes in mRNA signatures (Zaas et al., 2009, 2013; Mejias et al., 2013; Suarez et al., 2015). In contrast, the miRNA bacterial vs. viral classifier contains hs-miR-342-5p, known to be involved in the interferon antiviral response of macrophages against multiple viral pathogens (Robertson and Ghazal, 2016; Robertson et al., 2016). In our work, machine learning techniques identified diagnostic miRNA signatures that accurately differentiated patients with pneumococcal pneumonia from healthy subjects (accuracy 100%) or those with influenza infection (accuracy 91.3%) using a small number of miRNA probes (8 and 5, respectively). This small number of targets would facilitate translation to a clinically useful platform although highly multiplexed platforms are emerging that could potentially remove this barrier.

A true test of a biomarker’s ability to translate to clinical practice depends on whether it offers actionable results. The miRNA signatures presented here are highly sensitive for detection of *S. pneumoniae* bacterial infection (100% sensitivity in both models) compared to the 66.7% for the clinically available biomarker procalcitonin, suggesting a negative test could provide reassurance to avoid antibiotics when clinically appropriate. While this study is small and not representative of all ARI etiologies, these results hold promise that a miRNA signature could be a valuable tool in changing antibiotic prescribing practices.

For this pilot study, we analyzed circulating miRNA expression in whole blood. We chose to evaluate whole blood to support the goal of building miRNA:mRNA networks since our prior work on mRNA expression employed whole blood and samples from applicable retrospective cohorts were available. This is particularly noteworthy because reported miRNAs can regulate mRNA expression within the cell of origin and also act on distant tissues as secreted molecules (Gilad et al., 2008; Mitchell et al., 2008; Scholer et al., 2010). Similarly, miRNAs secreted from distant tissues could be present in the samples used for this analysis. The differences in miRNA expression reported here are most likely due to disease-specific effects. However, this pattern may also be affected by differing distributions of blood cell types and changes associated with comorbid conditions (Keller et al., 2011; Scholer et al., 2011) as well as variability in sampling, purification and other factors (Witwer, 2015). Further development of miRNA biomarkers for infection will need to evaluate the contributions of different fractions of blood (Keller et al., 2011; Scholer et al., 2011; Witwer, 2015) and employ controls that encompass comorbid conditions similar to our prior work with diagnostic mRNA signatures (Tsalik et al., 2016).

### miRNA:mRNA Regulatory Networks of Signature miRNAs Provide Insight Into the Biologic Response to Infection

Investigation of the miRNA:mRNA regulatory interface revealed from our signatures identified miRNAs and mRNAs with well described roles in the inflammatory response, supporting the biological relevance of the derived miRNA host response signatures. This study also identified miRNAs with less well defined roles in immunity, potentially identifying new role for these miRNAs in the host’s response to infection.

Several studies have identified a role for miRNAs in the regulation of sepsis, many of which were DE in this study. miRNA-150 was an important member of our bacterial vs. healthy signature and was also DE in the viral vs. healthy cohort. miRNA-150 predicts survival in patients with sepsis (Vasilescu et al., 2009; Roderburg et al., 2013; How et al., 2015), operating through MyD88 regulation, NFκB signaling (Sari et al., 2014), and toll-like receptor pathways (Ghorpade et al., 2013). Our analysis revealed downregulation of miRNA-150 during bacterial infection may exert broad effects on metabolism (SLCA2A3/GLUT3), cytokine regulation (SOCS1), and cell signaling pathways (MAPK13, IPO8, ACVR1B, RNF146) consistent with the host response to bacterial infection.

The dysregulation of miRNA-150 in both bacterial and viral responses compared to healthy may argue for a role as a master regulator of immunity. miRNA-150 has previously been shown to be dysregulated in viral infections (Chen et al., 2012, 2014) and appears to be a prognostic biomarker for severe influenza A/H1N1 infection (Moran et al., 2015). Network analysis of miRNA-150 in viral infections identified an association with a key interferon pathway member, interferon-induced protein with tetratricopeptide repeats 5 (IFIT5). The manner in which miRNA-150 regulates the host’s bacterial and viral response is unclear since each type of infection induces distinct biological pathways. It is possible that miRNA-150 induces a shared biological response or perhaps other regulatory molecules provide the specificity needed to direct the host’s response to bacterial or viral infection.

miRNA-30a-5p and miRNA-199-5p were common to the *S. pneumoniae* bacterial vs. healthy and bacterial vs. influenza signatures. In both cases, these miRNAs were upregulated in subjects with bacterial infection. miRNA-30a-5p has been tied to inhibition of IL-6 signaling through regulation of the JAK/STAT pathways via the suppressor of cytokine signaling 3 gene (SOCS3) (Xu et al., 2017). Our analysis suggests suppressor of cytokine signaling 1 gene (SOCS1) may also be involved in this pathway. MiRNA-199b-5p has been shown to directly target the transcription factor HES1 and downregulate the Notch pathway (Garzia et al., 2009), which plays a large role in innate immunity and inflammation (Shang et al., 2016).

Another important regulator of the immune response is the let-7 family of miRNAs. The let-7 family was the first group of miRNAs described over two decades ago (Lee et al., 1993; Wightman et al., 1993; Pasquinelli et al., 2000; Lagos-Quintana et al., 2001; Lau et al., 2001; Lee and Ambros, 2001) and has broad roles in development, differentiation, and metabolism (Mondol and Pasquinelli, 2012; Thornton and Gregory, 2012). Studies on their role in human disease have primarily focused on oncogenesis (Balzeau et al., 2017) but a role in the immue system is increasingly being recognized. The let-7 family of miRNAs has been associated with regulation of toll-like receptor 4 (Chen et al., 2007), control of cytokine levels (Hu et al., 2009; Schulte et al., 2011; Swaminathan et al., 2012), and regulation of the NFκB pathway (Kumar et al., 2015) in response to infection. Let-7 miRNAs are also found to be dysregulated in sepsis (How et al., 2015), viral infections (Bakre et al., 2012; Chen et al., 2012; Zhao et al., 2015), and parasitic infections (Hu et al., 2010). In this study, let-7a, let-7b, let-7d, and let-7e were DE in the *S. pneumoniae* infection vs. healthy comparison. The let-7e miRNA:mRNA network revealed regulation of cellular transport processes associated with innate immunity and autophagy (GNS, KPNA4, WDFY3, GALNT2, and UBXN4). Our network analysis also further suggested an association of let-7e with regulation of the IL6-receptor, consistent with existing data that the family of let-7 miRNAs are directly involved in IL6 regulation (Iliopoulos et al., 2009; Schulte et al., 2011). These are consistent with the host response to bacterial pathogens.

The presence of these miRNAs (miRNA-30a-5p, miRNA-199-5p, miRNA-150, and let 7e) in our diagnostic signatures demonstrate a strong role of innate immunity in response to infection and discrimination of bacterial and viral infections. However, differences between the *S. pneumoniae* vs. healthy and *S. pneumoniae* vs. influenza miRNA signatures revealed evidence of a more specific response to bacterial and viral infection. For example, miRNA-342-5p and miRNA-503-5p represent a distinct viral response in the *S. pneumoniae* vs. influenza miRNA signature. These miRNAs are known regulators of the interferon pathway through sterol synthesis (Robertson and Ghazal, 2016; Robertson et al., 2016) and association with Interleukin-1 receptor-associated kinase 2 (*IRAK2*) (Sanchez-Jimenez et al., 2013; Zhang W. et al., 2014), respectively. Interestingly, our network analysis also identified a potential new role for miRNA-342 in cytoskeleton regulation (PFN1, PXN) during infection. While little is known about miRNA-942-5p, studies demonstrate differential expression in response to dengue infection compared to healthy patients (Ouyang et al., 2016), implying a role in the host response to viral infections. Conversely, the presence of miRNA-423 and miRNA-769 in the *S. pneumoniae* vs. healthy signature suggest an immune response unique to bacterial infection. MiRNA-423 is expressed in neutrophils (Landgraf et al., 2007; Ward et al., 2011) and this class of white blood cells is part of the classic innate immune response to bacterial pathogens. MiRNA-769 has been implicated in bacterial pulmonary tuberculosis (Fu et al., 2014; Wu et al., 2014) although our results suggested a possibly larger role in bacterial infection more generally.

Two miRNAs were identified in our *S. pneumoniae* vs. healthy signature that did not previously have identified roles in regulating the immune system. miRNA-5189 is expressed in platelets (Ple et al., 2012) and is associated with lymphoblastic leukemias (Schotte et al., 2011). miRNA-2355 has not been identified in other biological processes.

### Limitations

This pilot study was limited by its small cohort size and the need to pool subjects from two independent retrospective cohorts. This study also failed to identify a large number of DE miRNAs when comparing influenza infection to the healthy baseline state. This is in contrast to prior analyses of mRNA (Zaas et al., 2009, 2013; Woods et al., 2013) and miRNA expression (Song et al., 2013; Tambyah et al., 2013) where differential expression during influenza infection was observed. Human influenza challenge subjects available for this study demonstrated lower symptom scores and milder clinical disease than the severe, hospitalized patients in prior miRNA studies. Furthermore, miRNA regulation is a dynamic process and may differ depending on the timing of infection. We chose a single time point, that of maximal symptoms during the human challenge experiment. Earlier or later time points may have identified more robust changes in the miRNA response or perhaps different responses altogether. A more comprehensive analysis of miRNA expression throughout the entire experimental time series would address this limitation. Despite these limitations we were able to achieve robust statistical results for bacterial vs. viral comparison, recapitulating the known viral induced interferon response and incorporating miRNAs distinct to that comparison.

Another potential limitation is that the small sample size may have resulted in low signal amidst the noise of both biological and technical variability. This was mitigated by the choice of single pathogens: *S. pneumoniae* to represent bacterial infection and influenza H3N2 to represent viral infection. Whereas this might improve our ability to identify discriminating miRNAs, it limits generalizability. Therefore, validation in a more heterogeneous cohort will be required in the future.

The small cohort size also places the model at risk of overfitting. However, we observed mean RNA signature sizes within our LOOCV that agreed with our final signature sizes, suggesting that the model was capturing a more generalized representation of the underlying biology rather than being overly dependent on any particular test subject.

Derivation of host-response signatures are most robust when all clinically relevant groups are used, including non-infectious illness. In our study population, controls were healthy individuals without respiratory symptoms. Therefore, future work should include controls with non-infectious causes of acute respiratory symptoms, allowing a true determination of the host response to bacterial and viral infection independent of the host response to being ill.

## Conclusion

We report novel human-derived host-response miRNA signatures that accurately discriminate bacterial and viral ARIs. These signatures were derived by employing machine learning algorithms on miRNA data in conjunction with differential expression analysis of matched miRNA and mRNA data. Using an integrated transcriptomic approach, we were then able to show the putative biological underpinnings of the molecular signatures and make regulatory inferences of the host-response. Our work on miRNA adds to the multiple modalities available to generate a robust and discriminatory disease signature for viral and bacterial infections including mRNA and protein. These results offer new opportunities for diagnostic development and exploration of the host response to bacterial and viral infection.

## Availability of Data and Materials

Previously published mRNA array data from 31 subjects includes 231 CEL files that are publically available on the GEO server under IDs GSE63990 and GSE73072. There were 6 CEL files from 2 subjects who were not contained in these published data. The miRNA datasets generated and/or analyzed during the current study are not publicly available as they are pending invention disclosure and evaluation for patent. All data are available from the corresponding author on reasonable request.

## Ethics Statement

Studies were approved by relevant Institutional Review Boards (IRB) and in accordance with the Declaration of Helsinki. All subjects or their legally authorized representatives provided written informed consent. Specifically, the CAPSOD study was approved by the Duke IRB, the Durham Veteran’s Affairs Hospital IRB, University of North Carolina IRB, and the Henry Ford Hospital IRB. The Human Influenza Challenge Study were approved by the Duke IRB, the Western IRB, and the United Kingdoms Ethics Board. Both the CAPSOD study and the Human Influenza Challenge Study were sponsored by the Defense Advanced Research Projects Agency (DARPA) who reviewed all study protocols, IRB authorizations, and all study procedures and ultimately gave approval for the work to be done.

## Author Contributions

CW, GG, ET, TB, and EK envisioned the project. CW and ET were involved in the clinical specimen collection and the adjudication process. GP, KS, CH, AV, MN, EH, and RH performed the statistical analyses. EK, ET, GP, and AV wrote the manuscript. All authors read, revised, and approved the final manuscript.

## Conflict of Interest Statement

Unrelated to this study, ET has consulted for Immunexpress and bioMerieux and received grants from DARPA, NIH, Novartis Vaccines and Diagnostics, Inc., and the Henry M. Jackson Foundation. CW has consulted for bioMerieux, Nanosphere, Luminex, and Becton Dickinson and received grants from DARPA, the Defense Threat Reduction Agency, NIH, Novartis Vaccines and Diagnostics, Inc., and the Henry M. Jackson Foundation. ET, CW, and GG have patents pending for Biomarkers for the Molecular Classification of Bacterial Infection pending, and Methods to Diagnose and Treat Acute Respiratory Infections pending; and has equity stake in Host Response, Inc. The remaining authors declare that the research was conducted in the absence of any commercial or financial relationships that could be construed as a potential conflict of interest.

## Abbreviations

ARI: acute respiratory infection
AUC: area under the curve
CDC: Centers for Disease Control and Prevention
CI: confidence interval
DAVID: Database for Annotation, Visualization, and Integrated Discovery
DE: differentially expressed
ED: emergency department
FDR: false discovery rate
GO: gene ontology
LASSO: least absolute shrinkage and selection operator
LOOCV: leave-one-out-cross-validation
PCA: principal components analysis
PVCA: principal variance component analysis
*Q*-value: Benjamini–Hochberg adjusted *p*-value and synonymous with false discovery rate
RMA: robust microarray average method
ROC: receiver operator characteristic
SNM: supervised normalization of microarrays
