# Supplementary material for: A miRNA Host Response Signature Accurately Discriminates Acute Respiratory Infection Etiologies
Source: Front Microbiol. 2018 Dec 11;9:2957. doi: 10.3389/fmicb.2018.02957 (PMC6298190; doi:10.3389/fmicb.2018.02957)
Supplement: Supplementary file 1 [file Table_1.pdf]

Table S1. Differentially expressed miRNAs and respective p-values for bacterial versus healthy comparison.

miRNA	p-value	miRNA	p-value
hsa-let-7a-5p	3.39E-05	hsa-miR-29c-5p	1.25E-09
hsa-let-7a-5p.1	3.41E-05	hsa-miR-30a-5p	4.78E-07
hsa-let-7a-5p.2	3.42E-05	hsa-miR-30d-5p	4.16E-04
hsa-let-7b-5p	5.81E-04	hsa-miR-30e-5p	1.46E-04
hsa-let-7d-5p	1.82E-07	hsa-miR-3173-5p	8.31E-04
hsa-let-7e-5p	1.44E-04	hsa-miR-32-5p	1.44E-03
hsa-miR-10b-5p	9.63E-07	hsa-miR-3200-5p	3.25E-04
hsa-miR-1254	3.55E-04	hsa-miR-320b	3.06E-04
hsa-miR-1254.1	3.55E-04	hsa-miR-320c	8.51E-04
hsa-miR-1260a	2.76E-05	hsa-miR-324-5p	1.34E-05
hsa-miR-1260b	2.06E-05	hsa-miR-330-5p	6.55E-06
hsa-miR-1285-5p	8.25E-05	hsa-miR-342-5p	8.43E-15
hsa-miR-1306-5p	2.82E-04	hsa-miR-345-5p	3.61E-05
hsa-miR-130b-5p	3.01E-05	hsa-miR-3614-5p	5.09E-06
hsa-miR-140-5p	1.39E-03	hsa-miR-362-5p	3.93E-05
hsa-miR-145-5p	2.42E-09	hsa-miR-374b-5p	1.24E-06
hsa-miR-146b-5p	1.40E-06	hsa-miR-3944-5p	1.96E-03
hsa-miR-148a-5p	6.29E-12	hsa-miR-423-5p	5.14E-11
hsa-miR-148b-5p	2.10E-04	hsa-miR-451a	7.37E-04
hsa-miR-150-5p	1.34E-06	hsa-miR-4732-5p	6.73E-09
hsa-miR-186-5p	2.78E-07	hsa-miR-4746-5p	9.84E-07
hsa-miR-192-5p	3.60E-04	hsa-miR-4772-5p	1.15E-04
hsa-miR-194-5p	7.56E-06	hsa-miR-484	3.21E-07
hsa-miR-194-5p.1	5.12E-06	hsa-miR-5010-5p	1.38E-04
hsa-miR-1976	2.05E-04	hsa-miR-503-5p	8.03E-06
hsa-miR-199b-5p	2.14E-23	hsa-miR-532-5p	5.39E-04
hsa-miR-21-5p	3.31E-09	hsa-miR-5690	1.29E-05
hsa-miR-210-5p	1.39E-08	hsa-miR-584-5p	1.07E-07
hsa-miR-2110	1.66E-04	hsa-miR-618	1.17E-04
hsa-miR-2355-5p	7.79E-05	hsa-miR-6502-5p	2.58E-05
hsa-miR-26a-5p	1.44E-08	hsa-miR-660-5p	2.12E-07
hsa-miR-26a-5p.1	1.43E-08	hsa-miR-769-5p	1.62E-15
hsa-miR-27a-5p	7.40E-05	hsa-miR-942-5p	6.90E-06
hsa-miR-296-5p	1.91E-03	hsa-miR-99a-5p	3.40E-04
