# Supplementary material for: A miRNA Host Response Signature Accurately Discriminates Acute Respiratory Infection Etiologies
Source: Front Microbiol. 2018 Dec 11;9:2957. doi: 10.3389/fmicb.2018.02957 (PMC6298190; doi:10.3389/fmicb.2018.02957)
Supplement: Supplementary file 2 [file Table_2.pdf]

Table S2. Differentially expressed miRNAs and respective p-values for bacterial versus viral comparison.

miRNA	pvalue	miRNA	pvalue
hsa-miR-10b-5p	4.42E-04	hsa-miR-26a-5p	3.39E-07
hsa-miR-1254	2.64E-05	hsa-miR-26a-5p.1	3.36E-07
hsa-miR-1254.1	2.64E-05	hsa-miR-29c-5p	2.61E-06
hsa-miR-1260a	5.65E-04	hsa-miR-30a-5p	7.00E-06
hsa-miR-1260b	5.59E-04	hsa-miR-3200-5p	7.55E-04
hsa-miR-1285-5p	1.65E-03	hsa-miR-324-5p	1.68E-04
hsa-miR-1306-5p	1.05E-03	hsa-miR-330-5p	2.27E-04
hsa-miR-130b-5p	1.21E-03	hsa-miR-342-5p	3.52E-09
hsa-miR-142-5p	6.67E-04	hsa-miR-362-5p	5.39E-04
hsa-miR-145-5p	2.21E-09	hsa-miR-374b-5p	2.19E-06
hsa-miR-146b-5p	7.64E-08	hsa-miR-423-5p	5.72E-07
hsa-miR-148a-5p	1.10E-09	hsa-miR-4732-5p	9.91E-07
hsa-miR-148b-5p	7.40E-04	hsa-miR-4746-5p	2.11E-05
hsa-miR-186-5p	1.70E-03	hsa-miR-484	3.41E-07
hsa-miR-194-5p	1.63E-03	hsa-miR-5010-5p	1.91E-04
hsa-miR-194-5p.1	1.17E-03	hsa-miR-503-5p	1.53E-06
hsa-miR-1976	1.50E-04	hsa-miR-5690	1.45E-04
hsa-miR-199b-5p	1.57E-15	hsa-miR-584-5p	4.12E-06
hsa-miR-21-5p	1.16E-06	hsa-miR-769-5p	1.07E-12
hsa-miR-210-5p	1.04E-06	hsa-miR-942-5p	1.46E-05
hsa-miR-2110	5.51E-04		
