# Supplementary material for: A miRNA Host Response Signature Accurately Discriminates Acute Respiratory Infection Etiologies
Source: Front Microbiol. 2018 Dec 11;9:2957. doi: 10.3389/fmicb.2018.02957 (PMC6298190; doi:10.3389/fmicb.2018.02957)
Supplement: Supplementary file 3 [file Table_3.docx]

**Table S3:** Procalcitonin results for subjects with *S. pneumoniae*, where residual sample was available. For reference, values >0.25 µg/liter are typically used to indicate the presence of a bacterial infection.

| Subject | PCT value |
| --- | --- |
| S.pneumo #1 | 0.2146 |
| S.pneumo #2 | 26.9300 |
| S.pneumo #3 | 4.1280 |
| S.pneumo #4 | 0.1517 |
| S.pneumo #5 | 56.8 |
| S.pneumo #6 | not available |
| S.pneumo #7 | 14.52 |
| S.pneumo #8 | 0.9441 |
| S.pneumo #9 | 1.191 |
| S.pneumo #10 | 0.0524 |
